# Intercropping Alters the Soil Microbial Diversity and Community to Facilitate Nitrogen Assimilation: A Potential Mechanism for Increasing Proso Millet Grain Yield

**DOI:** 10.3389/fmicb.2020.601054

**Published:** 2020-11-24

**Authors:** Ke Dang, Xiangwei Gong, Guan Zhao, Honglu Wang, Aliaksandr Ivanistau, Baili Feng

**Affiliations:** ^1^College of Agronomy, State Key Laboratory of Crop Stress Biology in Arid Areas/Northwest A & F University, Yangling, China; ^2^Belarusian State Agricultural Academy, Gorki, Belarus

**Keywords:** cereal–legume intercropping, soil microbial community, soil–plant system, N availability, grain yield

## Abstract

Intercropping of cereals and legumes has been used in modern agricultural systems, and the soil microorganisms associated with legumes play a vital role in organic matter decomposition and nitrogen (N) fixation. This study investigated the effect of intercropping on the rhizosphere soil microbial composition and structure and how this interaction affects N absorption and utilization by plants to improve crop productivity. Experiments were conducted to analyze the rhizosphere soil microbial diversity and the relationship between microbial composition and N assimilation by proso millet (*Panicum miliaceum* L.) and mung bean (*Vigna radiata* L.) from 2017 to 2019. Four different intercropping row arrangements were evaluated, and individual plantings of proso millet and mung bean were used as controls. Microbial diversity and community composition were determined through Illumina sequencing of 16S rRNA and internal transcribed spacer (ITS) genes. The results indicated that intercropping increased N levels in the soil–plant system and this alteration was strongly dependent on changes in the microbial (bacterial and fungal) diversities and communities. The increase in bacterial alpha diversity and changes in unique operational taxonomic unit (OTU) numbers increased the soil N availability and plant N accumulation. Certain bacterial taxa (such as Proteobacteria) and fungal taxa (such as Ascomycota) were significantly altered under intercropping and showed positive responses to increased N assimilation. The average grain yield of intercropped proso millet increased by 13.9–50.1% compared to that of monoculture proso millet. Our data clearly showed that intercropping proso millet with mung bean altered the rhizosphere soil microbial diversity and community composition; thus, this intercropping system represents a potential mechanism for promoting N assimilation and increasing grain yield.

## Introduction

With the increasing global demand for food, the relationship between crop production and food security should be determined and natural resources must be preserved (Banik and Sharma, 2009). Although industrial agriculture is directly beneficial to improving labor efficiency and crop production, intense fertilizer use has led to a series of ecological environmental problems, such as loss of diversity in ecosystems, decreases in soil fertility and aggravation of environmental pollution (Boardman et al., 2003; Jacobsen et al., 2013). Intercropping is a useful agricultural practice that permits the simultaneous growth of two or more crops in the same field, thereby improving the land use efficiency (Yu et al., 2017). This agricultural practice is a technological method based on the ecological principles of facilitation and complementarity (Duchene et al., 2017). Thus, intercropping has not only been adopted by developing countries but also European countries (Martin-Guay et al., 2017). Among different intercropping combinations, cereal–legume intercropping systems have become sustainable farming models because these types of crops are not competing for the same niche (Li et al., 2001) and have different nitrogen (N) use abilities or obtain N via different pathways (e.g., by mineral or organic fertilizer in cereals and N fixation in legumes) (Ghosh et al., 2009). Legumes reduce N input requirements by biological N fixation, which meets 50–60% of the N demand (Salvagiotti et al., 2008). Several studies have shown that intercropped crops use soil nutrients more efficiently than monocultured crops because of the higher recovery of N, increased yields of dry matter, and lack of negative impacts on the environment (Inal et al., 2007; Luo et al., 2016). Efficient utilization of N in belowground plant parts can promote the optimal growth of aboveground plant parts. Thus, the roles of effective planting patterns as a means of maintaining N supplies are valuable in modern agriculture.

Soil microorganisms account for a large part of the earth’s biodiversity and play a significant role in soil ecosystem biochemical processes, such as nutrient cycling and soil–borne pathogen suppression (Gomes et al., 2003; Zhang et al., 2018). Microbial diversity and composition are key determinants of their ecological functions. Most of these N-fixing microorganisms (diazotrophs) exist in free-living conditions and provide an average of 110 million tons of N per year to terrestrial ecosystems (Xu et al., 2019). In cereal–legume intercropping systems, N fixation by microorganisms associated with legumes increases the availability of N for plants, with soil N uptake by intercropped cereal greatly inducing N fixation by the root nodules in the legume rhizosphere, which stimulates N utilization by coordinating interspecific interactions (Latati et al., 2016). This practical technique influences N transformation processes by regulating microbial activities, diversities and community compositions (Lian et al., 2019; Vidal et al., 2019). Actinobacteria and Proteobacteria, two common and ubiquitous bacterial groups, are markedly affected by intercropping systems due to their biology and habitats (Stach and Bull, 2005; Gong et al., 2019b). Most N-fixing microorganisms belong to Proteobacteria and are aerobic organisms. Li et al. (2016) proposed that below-surface interactions in an maize/peanut intercropping system considerably altered the microbial structure of the soil and the dominant microbial species that are strongly linked to soil N availability. Chen et al. (2018) revealed that peanut/maize intercropping promotes plant N uptake and increases the abundance of N-cycling organisms and other beneficial rhizosphere bacteria. Lian et al. (2019) demonstrated that sugarcane/soybean intercropping in acidic soil increases microbial diversity and shifts soil microbial communities, which may stimulate N accumulation by the legume. Therefore, reasonable cereal–legume intercropping boosts soil N conversion by improving soil microbial activities and increases soil available N contents (Zuo and Zhang, 2009).

Proso millet (*Panicum miliaceum* L.) is one of the earliest cultivated crops in China, and it has a short growing season and is primarily planted as an important food source in the semiarid regions of China because of its high nutritional value and strong resistance to drought, saline-alkaline conditions and poor soil conditions (Seghatoleslami et al., 2008; Yang et al., 2018). Mung bean (*Vigna radiata* L.) is regarded as a functional food (Yao et al., 2019), and its functional components have been extracted and identified using analytical techniques. Mung bean also has a separate benefit as it engages in symbiosis with N–fixing bacteria (Choudhary and Agrawal, 2014). The intercropping combination of proso millet and mung bean has been regarded as a technically sound method based on the ecological principles of facilitation and complementarity. Thus, this practice has been rapidly applied in Northwest China (Gong et al., 2019a, 2020a, 2020b). To date, although field experiments have been conducted to evaluate the impacts of cereal–legume intercropping in recent years (Latati et al., 2016; Cao et al., 2017; Qian et al., 2018), information about crop productivity, soil microbial–mediated processes and N assimilation under proso millet–mung bean intercropping systems is still limited. Thus, we hypothesized that proso millet intercropped with mung bean will increase the soil microbial diversity, regulate the microbial community composition and efficiently contribute to N uptake and assimilation to obtain high grain yields.

The main purposes of this research were to (i) investigate the impacts of intercropping on the microbial diversity and community composition of proso millet rhizosphere soil and compare bacterial and fungal reactions to intercropping systems; (ii) explore N accumulation in soil and different plant organs and the changes in grain yield under intercropping systems; and (iii) analyze the potential microbial groups that contribute to N retention and increase grain yield. This study can provide insights into the fundamental processes of biodiversity enhancement in ecosystems for developing sustainable agriculture.

## Materials and Methods

### Experimental Locations

Field experiments were conducted from 2017 to 2019 at Northwest A&F University’s experimental site (37°56′26″N, 109°21′46″E) in Yulin City, Shaanxi Province, China (Supplementary Figure 1a). This area is characterized by a semiarid continental monsoon climate, and the precipitation and annual average temperature in the area are 400 mm and 8.3°C, respectively. The daily air temperature and precipitation during the crop growing seasons (May-September) in 2017, 2018, and 2019 are shown in Supplementary Figure 2. The soil pH was 8.6, the organic matter content was 7.34 g kg^–1^, the total N (TN) was 0.36 g kg^–1^, the total phosphorus (P) was 0.75 g kg^–1^, and the total potassium (K) was 17.88 g kg^–1^ of dry soil in the 0–20 cm soil layer before sowing.

### Experimental Design and Treatments

The experimental design of this study was the same as that described in Gong et al. (2020a, b), and it consisted of a randomized complete block with four replicates. Four different intercropping patterns were designed as follows: 2 rows of proso millet intercropped with 2 rows of mung bean (2P2M), 4 rows of proso millet intercropped with 2 rows of mung bean (4P2M), 4 rows of proso millet intercropped with 4 rows of mung bean (4P4M), and 2 rows of proso millet intercropped with 4 rows of mung bean (2P4M). Monoculture proso millet (MP) and mung bean (MM) were set as controls (Supplementary Figure 1b). All in–row distances were 0.33 m, and each experimental plot had an area of 30 m^2^ (6 × 5 m) and included at least three strips of proso millet and mung bean. Border rows were not used for sampling.

The cultivars ‘Shanmi–1’ (proso millet) and ‘Zhonglv–8’ (mung bean) were selected for use in this study. Proso millet was sown on 12 June and harvested on 23 September 2017, sown on 10 June and harvested on 25 September 2018, and sown on 10 June and harvested on 30 September 2019. Mung bean was sown on 28 May and harvested on 24 August 2017, sown on 18 May and harvested on 20 August 2018, and sown on 25 May and harvested on 30 August 2019. The previous season’s crops were yam, maize and potato for 2017, 2018, and 2019, respectively. The plant densities of proso millet and mung bean were 50 plants m^–2^ and 20 plants m^–2^, respectively, whereas the densities of both the monoculture crops and intercrops were the same. Basal fertilizers [120 kg (N) ha^–1^, 100 kg (P_2_O_5_) ha^–1^, and 75 kg (K_2_O) ha^–1^] were applied to the soils before planting (proso millet and mung bean) each year. During the growth period, fertilizers were not applied.

### Plant Sampling and Grain Yield

Plants were sampled at the flowering stage of proso millet and at the filling stage of mung bean from 2017 to 2019. Three plants were randomly selected from the center of each plot (for a total of 12 plants) and split into stem, leaf, sheath and ear samples for proso millet and into stem, leaf, petiole and pod samples for mung bean. All aboveground samples were dried at 75°C until reaching a constant weight. The N content of different organs was measured by the Kjeldahl method after digestion with H_2_SO_4_–H_2_O_2_. At harvest, twenty proso millet plants were selected randomly in each plot (5 plants per treatment) to measure the ear length, ear number per plant, grain weight per plant and 1000-grain weight for proso millet. Similarly, the branch number per plant, pod number per plant, grain weight per plant and 100-grain weight for mung bean were investigated following the same sampling method. All the plants were harvested in each plot, and the grain yield was determined by weighing after air-drying for 2–3 weeks.

### Soil Sampling and Analysis

Each soil sample was obtained from the rhizosphere of each intercropping pattern at the flowering stage of proso millet (60 days after sowing) and at the filling stage of mung bean (75 days after sowing) in August 2018 and 2019, respectively. Plants in each plot were selected using an ‘S’–shaped pattern and then homogenized to provide one composite sample per replicated site. During this sampling process, sterile paper was used to wipe the remains that were attached to the spade and sanitize the spade before collecting the next soil sample to avoid contamination between treatments and keep samples fresh. After the roots were gently shaken, the rhizosphere soils tightly attached to roots were collected and sieved through 2-mm mesh to remove stones and other residues. A portion of each soil sample was air-dried and used in the TN analysis, and another portion was stored at 4°C to determine the other N fractions. Subsamples for the molecular analyses were immediately homogenized in liquid nitrogen and stored at −80°C. Soil TN was measured as described by Li et al. (2016). Nitrate (NO_3_^–^–N) and ammonium (NH_4_^+^–N) levels were analyzed based on standard methods using a continuous flow analyzer (Yu et al., 2019). Soil microbial biomass N (MBN) was determined using a chloroform fumigation–extraction method (Zhao et al., 2018). Four soil samples were analyzed per treatment.

### Soil DNA Extraction, PCR Amplification and Sequencing Analysis

Microbial DNA of fresh rhizosphere soil (0.5 g) of proso millet was extracted four times (total of 2.0 g soil). The genomic DNA concentration and quality were estimated by 1.0% Sepharose gels. The bacterial 16S rRNA gene V3–V4 hypervariable region was amplified with primers 338F (ACTCCTACGGGAGGCAGCAG) and 806R (GGACTACHVGGGTWTCTAAT) (Zhang et al., 2015). A 10–digit barcode sequence was attached to the 5′ end of the forward and reverse primers in every soil sample (provided by Auwigene Company, Beijing). The PCR mixture included 4 μL of 12.5 Mm dNTP Mix, 5 μL of 10 × Ex Taq Buffer (Mg^2+^ plus), 200 nm of barcoded primers 16S–F and 16S–R, 1.25 U Ex Taq DNA polymerase, 2 μL of template DNA and 36.75 μL of ddH_2_O. The following procedure was used for PCR: an initial denaturation step for 2 min at 94°C, followed by 30 cycles of 94°C for 30 s, 57°C for 30 s and 72°C for 30 s, and a final extraction at 72°C for 5 min. The fungal internal transcribed spacer (ITS) region was amplified on an Eppendorf Mastercycler Gradient Thermocycler (Germany) with the primers ITS1F (5-CTTGGTCATTTAGAGGAAGTAA-3) and ITS2 (5-TGCGTTCTTCATCGATGC-3) (Wang et al., 2016). The 5′ ends of the two primers were tagged. Ultra–PAGE purified primers were obtained from Majorbio, China. The PCR mixture included 4 μl of 5 × FastPfu Buffer, 2.5 Mm dNTP mixture, 5 μM each primer, 2 μl of template DNA and 10 μl of H_2_O. The following procedure was used for PCR: an initial denaturation step for 2 min at 95°C, followed by 30 cycles of 95°C for 30 s, 55°C for 30 s and 72°C for 30 s, and a final extraction at 72°C for 5 min. Deep sequencing of bacterial and fungal samples was performed on the MiSeq platform at Allwegene Company in Beijing, China. Sequence data associated with this project have been deposited into the National Center for Biotechnology Information (NCBI) (accession numbers of PRJNA669229 for bacteria and PRJNA669216 for fungi).

### Analysis of 16S rRNA and ITS Gene Data

For both bacterial and fungal reads, raw sequences were first trimmed and the reads were quality filtered, demultiplexed and processed on QIIME (Ren et al., 2018; Mcknight et al., 2019). Sequences were retained according to three criteria: (1) the barcodes and primers were explicit; (2) the length was greater than 200 bp; and (3) the quality score was higher than 30. All sequences were classified into different taxonomic groups by the Ribosomal Database Project classifier (Wang et al., 2007). The sequences, based on 97% similarity, were clustered into operational taxonomic units (OTUs) to produce rarefaction curves (Colwell and Coddington, 1994) and calculate the diversity and richness indices (Cole et al., 2008).

### Statistical Analyses

The taxonomic alpha diversity, which represents the community diversity, was calculated by the Shannon index using Mothur software (v.1.30.1). The taxonomic beta diversity (the weighted UniFrac distances) illustrates the clustering of different samples and reflecting the microbial community structure, and it was determined through principal coordinate analysis (PCoA). The correlations among plant properties, soil N and soil microbial compositions were determined by a redundancy analysis (RDA) using the CANOCO 5.0 software package. We implemented a forward selection procedure according to the method described by Blanchet et al. (2008) to select a subset of plant properties, soil N and soil microbial compositions. The data were analyzed via a one-way ANOVA (SPSS 19.0, SPSS Inc., Chicago, IL, United States) under different intercropping patterns. The relationships among microbial diversity, plant properties and soil N were determined via Spearman’s correlation analysis. The differences between mean values were determined using the least significant difference (LSD) test (*P* < 0.05), as indicated by different letters. Moreover, the ANOVA was conducted with the standard design analysis method to determine the significance of year and treatment effects and their interactions.

## Results

### Grain Yield

The grain yield of proso millet under the intercropping patterns significantly increased over the three years studied (Table 1). Across all treatments and years, intercropping significantly increased the ear number per plant, ear length, grain weight per plant and 1000-grain weight compared with monoculture proso millet and resulted in grain yield improvements of 5.6–20.7% in 2017, 7.9–53.9% in 2018, and 28.3–75.4% in 2019. Among the different intercropping systems, the 2P4M treatment achieved the greatest grain yield. Compared with the monoculture mung bean, the grain yield of intercropped mung bean was 34.8–55.8% lower in 2017, 34.4–38.8% lower in 2018, and 26.5–46.5% lower in 2019 (Table 2). The maximum and minimum reductions occurred in the 4P2M and 2P4M treatments, respectively.

**TABLE 1:** Effect of intercropping on grain yield and yield components of proso millet in 2017, 2018, and 2019.

Year	Treatment	Ear number (No. plant^–1^)	Ear length (cm)	Grain weight (g plant^–1^)	1000-grain weight (g)	Grain yield (kg ha^–1^)
2017	MP	4.0 ± 0.7d	39.2 ± 1.3cd	24.0 ± 1.9e	8.6 ± 10.0d	4448.6 ± 135.5d
	2P2M	4.8 ± 0.8bc	41.2 ± 2.1c	35.3 ± 1.1c	8.8 ± 90.1bc	4968.9 ± 87.2bc
	4P2M	4.3 ± 0.4c	40.1 ± 1.0c	32.5 ± 1.6d	8.8 ± 10.0c	4696.2 ± 76.8c
	4P4M	4.9 ± 0.2b	42.2 ± 0.8b	40.9 ± 0.4b	8.9 ± 20.1b	5131.6 ± 73.5b
	2P4M	5.8 ± 0.4a	44.0 ± 1.8a	46.6 ± 2.5a	9.0 ± 20.0a	5367.8 ± 56.8a
2018	MP	3.4 ± 0.9b	46.4 ± 2.2a	29.0 ± 1.3d	8.6 ± 60.1c	4205.7 ± 257.7d
	2P2M	4.6 ± 2.0ab	47.0 ± 2.5a	41.8 ± 0.8b	9.0 ± 00.0b	5153.8 ± 150.7b
	4P2M	4.4 ± 1.1ab	46.6 ± 2.6a	36.7 ± 0.7c	9.0 ± 30.1ab	4539.8 ± 144.6c
	4P4M	5.2 ± 1.5ab	47.2 ± 1.9a	43.2 ± 0.7b	9.1 ± 00.1ab	5249.6 ± 147.3b
	2P4M	6.2 ± 1.1a	47.2 ± 1.6a	50.4 ± 1.2a	9.1 ± 80.0a	6471.2 ± 236.6a
2019	MP	3.5 ± 0.6a	35.8 ± 2.8c	27.5 ± 2.7d	8.3 ± 90.2a	4162.9 ± 404.7d
	2P2M	4.5 ± 1.0a	41.2 ± 3.0b	42.5 ± 4.4b	8.4 ± 90.1a	6431.8 ± 659.9b
	4P2M	4.5 ± 0.6a	42.8 ± 1.5ab	35.3 ± 1.7c	8.4 ± 10.2a	5340.9 ± 252.5c
	4P4M	4.3 ± 0.5a	43.0 ± 4.1ab	45.5 ± 4.0ab	8.6 ± 20.2a	6890.2 ± 607.9ab
	2P4M	4.8 ± 1.7a	46.3 ± 1.9a	48.2 ± 2.2a	8.6 ± 60.1a	7303.0 ± 332.9a
**Variation source**						
Year		ns	**	**	**	*
Treatment		**	*	**	**	*
Year × Treatment		ns	ns	ns	ns	ns

Date are expressed as the means ± SE (*n* = 20). Values followed by different letters within a column are significantly different at *P* < 0.05. MP, 2P2M, 4P2M, 4P4M, and 2P4M represent the monoculture proso millet, 2 rows of proso millet intercropped with 2 rows of mung bean (2P2M), 4 rows of proso millet intercropped with 2 rows of mung bean (4P2M), 4 rows of proso millet intercropped with 4 rows of mung bean (4P4M), and 2 rows of proso millet intercropped with 4 rows of mung bean (2P4M), respectively. * and ** indicate significance at the 0.05 and 0.01 probability levels, respectively. ns, no significant difference.

**TABLE 2:** Effect of intercropping on grain yield and yield components of mung bean in 2017, 2018, and 2019.

Year	Treatment	Branch number (No. plant^–1^)	Pods number (No. plant^–1^)	Grain weight (g plant^–1^)	100-grain weight (g)	Grain yield (kg ha^–1^)
2017	MM	14.3 ± 2.2a	32.4 ± 1.8a	11.6 ± 0.7a	6.3 ± 90.7a	1297.3 ± 140.3a
	2P2M	8.6 ± 1.8cd	22.1 ± 1.7c	4.9 ± 0.3d	6.0 ± 11.3a	607.2 ± 38.8d
	4P2M	7.6 ± 1.2d	19.8 ± 1.5d	4.2 ± 0.3e	5.9 ± 50.6a	573.1 ± 79.6e
	4P4M	10.6 ± 0.7bc	23.7 ± 1.1c	6.0 ± 0.2c	6.1 ± 10.6a	722.3 ± 67.5c
	2P4M	11.9 ± 0.4b	27.8 ± 1.3b	7.3 ± 0.2b	6.2 ± 30.4a	845.5 ± 43.7b
2018	MM	15.3 ± 2.4a	52.3 ± 9.6a	16.3 ± 1.8a	7.1 ± 30.2a	1483.7 ± 36.5a
	2P2M	11.8 ± 1.7b	35.3 ± 8.8b	10.0 ± 1.0bc	6.9 ± 90.3ab	920.5 ± 56.3b
	4P2M	10.8 ± 1.5b	31.5 ± 11.7b	8.6 ± 1.2c	6.3 ± 00.2b	908.3 ± 64.0b
	4P4M	12.5 ± 1.7ab	37.8 ± 4.1b	10.3 ± 1.5bc	6.4 ± 10.6ab	957.6 ± 59.5b
	2P4M	12.0 ± 2.8ab	42.8 ± 5.9ab	11.2 ± 2.2b	6.6 ± 50.8ab	973.5 ± 57.6b
2019	MM	6.0 ± 0.8a	44.3 ± 9.0a	10.4 ± 2.1a	6.8 ± 50.1a	1326.1 ± 167.2a
	2P2M	4.5 ± 1.0b	34.5 ± 5.3ab	7.0 ± 1.0b	6.3 ± 90.1bc	709.6 ± 102.7c
	4P2M	4.8 ± 0.5ab	25.5 ± 4.4b	7.4 ± 2.1b	6.2 ± 20.2c	715.8 ± 105.9c
	4P4M	5.5 ± 1.0ab	31.0 ± 11.6b	8.7 ± 0.6ab	6.5 ± 20.2b	974.4 ± 19.4b
	2P4M	6.0 ± 0.8a	37.3 ± 6.8ab	6.9 ± 1.4b	6.5 ± 60.1b	731.6 ± 124.8c
**Variation source**						
Year		ns	**	**	ns	**
Treatment		**	**	**	ns	**
Year × Treatment		ns	ns	ns	ns	*

Date are expressed as the means ± SE (*n* = 20). Values followed by different letters within a column are significantly different at *P* < 0.05. MM, 2P2M, 4P2M, 4P4M, and 2P4M represent the monoculture mung bean, 2 rows of proso millet intercropped with 2 rows of mung bean (2P2M), 4 rows of proso millet intercropped with 2 rows of mung bean (4P2M), 4 rows of proso millet intercropped with 4 rows of mung bean (4P4M), and 2 rows of proso millet intercropped with 4 rows of mung bean (2P4M), respectively. * and ** indicate significance at the 0.05 and 0.01 probability levels, respectively. ns, no significant difference.

### N Levels in the Soil-Plant System

**FIGURE 1:**
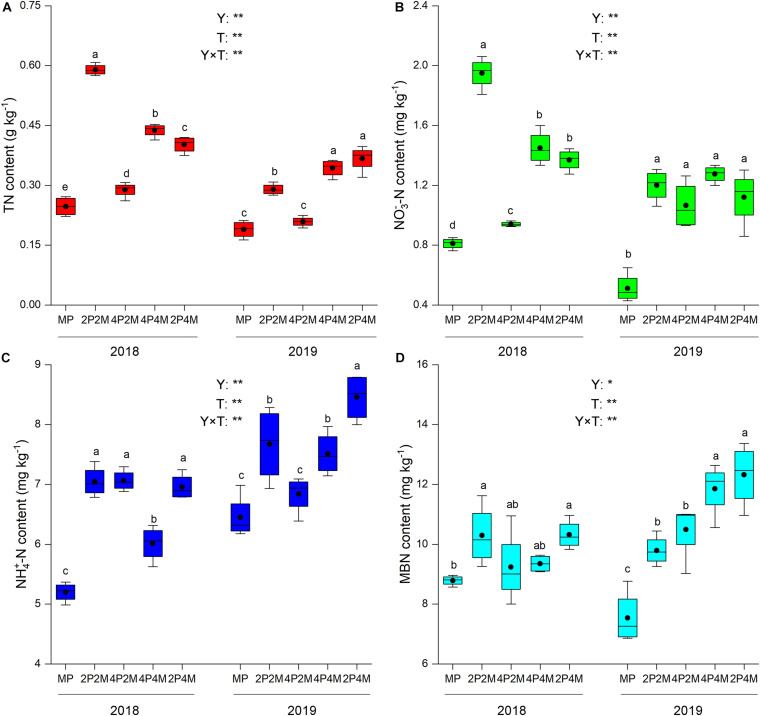
Effect of intercropping patterns on soil TN **(A)**, NO_3_^–^-N **(B)** NH_4_^+^–N **(C)**, MBN **(D)** of proso millet. Different letters indicate significant differences (*P* < 0.05) among different intercropping patterns. Total nitrogen (TN), nitrate (NO_3_^–^-N), ammonium (NH_4_^+^-N), microbial biomass nitrogen (MBN). MP, 2P2M, 4P2M, 4P4M, and 2P4M represent the monoculture proso millet, 2 rows of proso millet intercropped with 2 rows of mung bean (2P2M), 4 rows of proso millet intercropped with 2 rows of mung bean (4P2M), 4 rows of proso millet intercropped with 4 rows of mung bean (4P4M), and 2 rows of proso millet intercropped with 4 rows of mung bean (2P4M), respectively. * and ** Significant at the 0.05 and 0.01 probability levels, respectively. ns, no significant difference.

**FIGURE 2:**
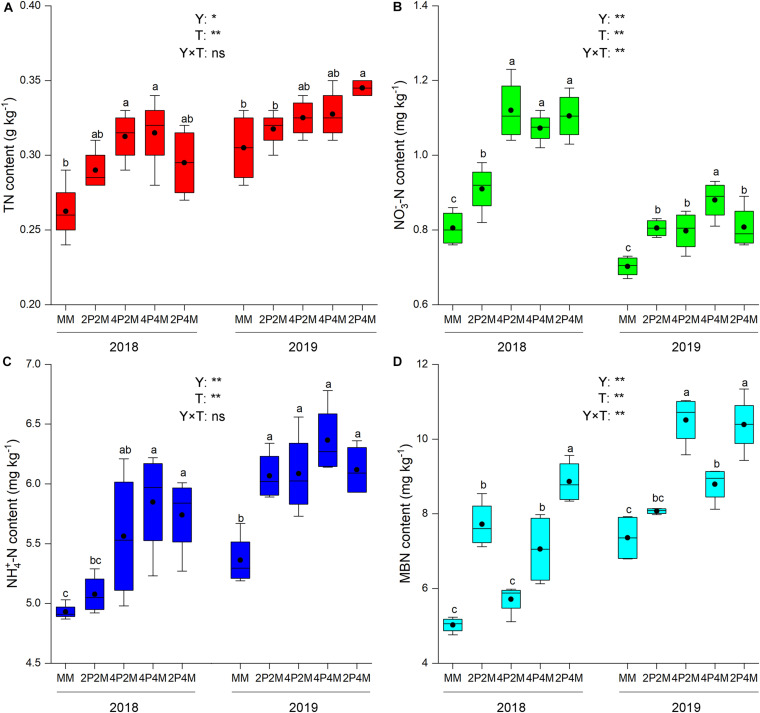
Effect of intercropping patterns on soil TN **(A)**, NO_3_^–^-N **(B)** NH4^+^–N **(C)**, MBN **(D)** of mung bean. Different letters indicate significant differences (*P* < 0.05) among different intercropping patterns. Total nitrogen (TN), nitrate (NO_3_^–^-N), ammonium (NH_4_^+^-N), microbial biomass nitrogen (MBN). MM, 2P2M, 4P2M, 4P4M, and 2P4M represent the monoculture mung bean, 2 rows of proso millet intercropped with 2 rows of mung bean (2P2M), 4 rows of proso millet intercropped with 2 rows of mung bean (4P2M), 4 rows of proso millet intercropped with 4 rows of mung bean (4P4M), and 2 rows of proso millet intercropped with 4 rows of mung bean (2P4M), respectively. * and ** Significant at the 0.05 and 0.01 probability levels, respectively. ns, no significant difference.

The differences in the soil-plant system between the proso millet and mung bean strips are shown in Figures 1, 2 and Table 3. Compared with the monoculture proso millet, the average N content under the intercropping patterns in the stem, leaf, sheath, and ear tissues increased by 28.6, 15.2, 16.7, and 12.0%, respectively (Figure 1). Among the intercropping systems, the 2P4M treatment resulted in the greatest plant N improvement. Similarly, the intercropping systems achieved greater plant N contents in mung bean, which were 19.3, 13.0, 18.2, and 15.4% higher than that of the monoculture bean across all treatments and years (Figure 2).

**TABLE 3:** Effect of intercropping on plant N content of proso millet and mung bean in 2017, 2018, and 2019.

Year	Treatment	Proso millet				Mung bean			
		Stem	Leaf	Sheath	Ear	Stem	Leaf	Petiole	Pod
2017	MP (MM)	7.0 ± 50.56c	22.2 ± 70.83d	15.5 ± 31.16a	22.4 ± 11.11b	15.6 ± 51.01b	28.2 ± 10.32b	16.1 ± 60.85b	15.9 ± 20.71c
	2P2M	8.6 ± 30.97ab	26.8 ± 10.65c	14.7 ± 01.26ab	24.0 ± 61.07a	18.4 ± 30.83a	29.5 ± 70.99a	19.2 ± 50.98a	17.6 ± 70.74b
	4P2M	9.3 ± 30.82ab	24.6 ± 41.14d	13.8 ± 30.92bc	23.5 ± 60.84ab	18.9 ± 51.25a	30.0 ± 91.45a	17.9 ± 21.22b	18.3 ± 90.54a
	4P4M	8.5 ± 70.83b	28.6 ± 10.46b	12.5 ± 00.75c	24.7 ± 90.94a	18.7 ± 71.28a	30.3 ± 81.15a	17.8 ± 51.45b	18.7 ± 11.02a
	2P4M	9.8 ± 50.64a	31.4 ± 60.92a	12.7 ± 70.83c	24.9 ± 61.11a	19.1 ± 81.05a	29.8 ± 01.08a	18.7 ± 90.93ab	18.1 ± 90.87a
2018	MP (MM)	9.3 ± 20.37d	27.5 ± 51.29c	18.9 ± 21.40a	25.8 ± 41.91a	13.6 ± 10.71c	20.5 ± 31.19c	13.3 ± 60.93c	13.3 ± 00.84b
	2P2M	13.2 ± 90.67c	31.9 ± 51.63b	18.1 ± 81.73a	24.2 ± 91.43a	14.6 ± 71.47b	21.5 ± 11.02b	15.1 ± 10.76b	14.3 ± 80.58a
	4P2M	11.2 ± 50.94c	32.0 ± 31.23b	13.4 ± 22.56b	23.5 ± 62.37a	13.8 ± 20.94bc	22.4 ± 71.66ab	16.3 ± 71.34ab	15.1 ± 30.30a
	4P4M	9.3 ± 50.80d	28.0 ± 21.02c	14.4 ± 61.43b	26.2 ± 21.60a	16.6 ± 40.64ab	21.6 ± 51.51b	17.4 ± 01.29a	15.0 ± 00.45a
	2P4M	15.7 ± 61.29a	34.3 ± 31.69a	15.2 ± 72.14b	25.9 ± 50.69a	17.5 ± 71.29a	23.1 ± 51.27a	15.2 ± 90.65b	15.5 ± 30.78a
2019	MP (MM)	8.3 ± 21.21d	23.5 ± 52.42c	11.4 ± 20.86c	22.8 ± 42.33c	17.3 ± 70.86b	22.4 ± 20.87b	15.0 ± 70.98b	15.5 ± 00.37b
	2P2M	9.2 ± 90.27c	26.9 ± 52.86b	12.4 ± 60.34b	24.2 ± 92.75c	17.7 ± 01.37b	22.9 ± 70.90b	17.1 ± 11.03a	18.4 ± 41.83a
	4P2M	11.2 ± 51.64ab	30.0 ± 32.76a	12.2 ± 71.15b	24.5 ± 61.13c	19.2 ± 51.31a	23.9 ± 41.73b	17.6 ± 20.46a	19.1 ± 82.71a
	4P4M	9.3 ± 50.62dc	32.0 ± 22.38a	15.9 ± 20.86a	29.2 ± 20.64b	20.3 ± 11.14a	25.7 ± 91.81q	17.3 ± 92.31a	18.7 ± 30.74a
	2P4M	12.7 ± 60.89a	32.3 ± 32.72a	15.1 ± 81.33a	31.9 ± 52.33a	20.4 ± 30.47a	26.6 ± 01.58q	15.8 ± 41.18b	18.4 ± 81.56a
**Variation source**									
Year		*	ns	**	*	**	**	ns	ns
Treatment		**	**	*	**	**	**	*	*
Year × Treatment		ns	ns	*	ns	ns	*	ns	ns

Date are expressed as the means SE (*n* = 20). Values followed by different letters within a column are significantly different at *P* < 0.05. MP, MM, 2P2M, 4P2M, 4P4M, and 2P4M represent the monoculture proso millet, monoculture mung bean, 2 rows of proso millet intercropped with 2 rows of mung bean (2P2M), 4 rows of proso millet intercropped with 2 rows of mung bean (4P2M), 4 rows of proso millet intercropped with 4 rows of mung bean (4P4M), and 2 rows of proso millet intercropped with 4 rows of mung bean (2P4M), respectively. * and ** indicate significance at the 0.05 and 0.01 probability levels, respectively. ns, no significant difference.

Intercropping greatly affected the soil N contents of these two species over the two years but showed differential effects (Table 3). For proso millet, the average TN, NO_3_^–^–N, NH_4_^+^–N, and MBN were 67.7, 96.1, 23.5, and 28.1% higher under intercropping than monoculture, respectively. Among the intercropping patterns, 2P2M resulted in the maximum TN and NO_3_^–^–N, which increased by 101.6 and 138.3%, respectively, while 4P4M resulted in the highest improvement in NH_4_^+^–N and MBN, which increased by 32.3% and 38.7%, respectively, compared with the MP. For mung bean, TN, NO_3_^–^–N and NH_4_^+^–N were higher under intercropping than monoculture and the maximum values were achieved under the 4P4M treatment over the two years. Similarly, the large increase in MBN also corresponded to the intercropping system, and the 2P4M treatment achieved the maximum increase (55.5% higher than that under the MM treatment).

### Soil Microbial Diversity

**FIGURE 3:**
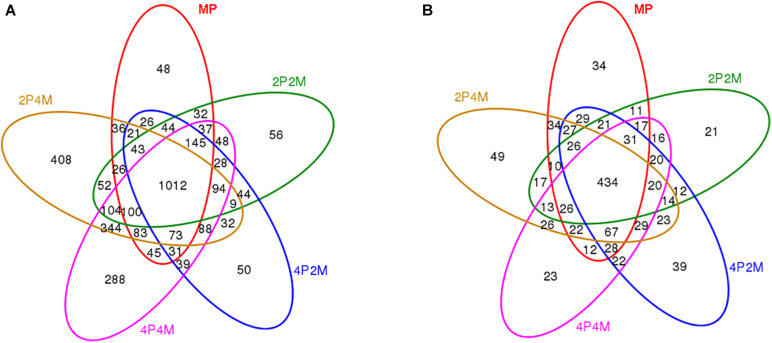
Venn diagram displaying the OTU richness distribution of bacteria **(A)** and fungi **(B)** in five soil treatments. MP, 2P2M, 4P2M, 4P4M, and 2P4M represent the monoculture proso millet, 2 rows of proso millet intercropped with 2 rows of mung bean (2P2M), 4 rows of proso millet intercropped with 2 rows of mung bean (4P2M), 4 rows of proso millet intercropped with 4 rows of mung bean (4P4M), and 2 rows of proso millet intercropped with 4 rows of mung bean (2P4M), respectively.

Venn graphs were constructed to evaluate the number and identity of the shared OTUs for proso millet from the five soil treatments (Figure 3). For bacteria, 1012 OTUs were jointly shared among the five treatments and 2P4M had the most OTUs that were specific to other areas (Figure 3A). For fungi, only 434 OTUs were common to the different treatments and specific OTUs increased under 4P2M and 2P4M and decreased under 2P2M and 4P4M compared with those under MP (Figure 3A).

**FIGURE 4:**
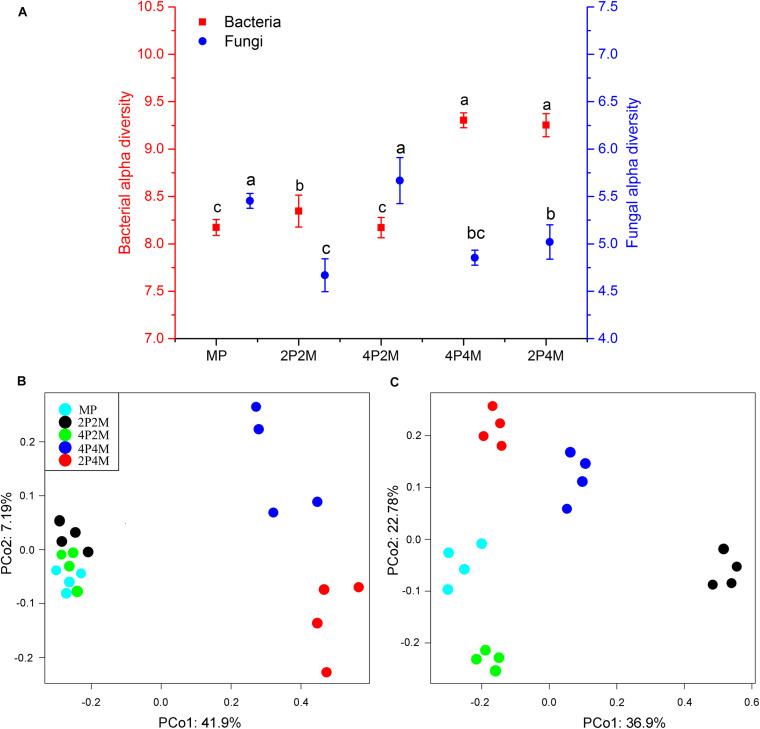
Effect of intercropping patterns on the soil microbial alpha diversity (Shannon index) **(A)** and beta diversity of proso millet (principle coordinates analysis, PCoA) (bacterial, **B** and fungal, **C**). Different letters indicate significant differences (*P* < 0.05) among different intercropping patterns. MP, 2P2M, 4P2M, 4P4M, and 2P4M represent the monoculture proso millet, 2 rows of proso millet intercropped with 2 rows of mung bean (2P2M), 4 rows of proso millet intercropped with 2 rows of mung bean (4P2M), 4 rows of proso millet intercropped with 4 rows of mung bean (4P4M), and 2 rows of proso millet intercropped with 4 rows of mung bean (2P4M), respectively.

Microbial diversity under the different planting patterns was represented using an OTU-level approach. Figure 4A shows that intercropping clearly altered the soil bacterial alpha diversity (*P* < 0.01). The 4P4M treatment showed the highest diversity, and the MP treatment showed the lowest, with values ranging from 8.17 to 9.30. The Shannon index results displayed different changes for fungi, and the value only increased under 4P2M and decreased under 2P2M, 4P4M and 2P4M. To visualize and determine the similarities in the species composition data, the effects of different intercropping patterns on microbial community beta diversity were determined via a PCoA (Figures 4B,C). The soil bacterial communities in the 4P4M and 2P4M treatments (4 replicates of each treatment) were different from those in the 2P2M, 4P2M and MP treatments (Figure 4B). In contrast, the fungal community composition of the intercropping soil was very different from that of the MP soil, and a similar change trend was not observed (Figure 4C).

### Soil Microbial Composition

**FIGURE 5:**
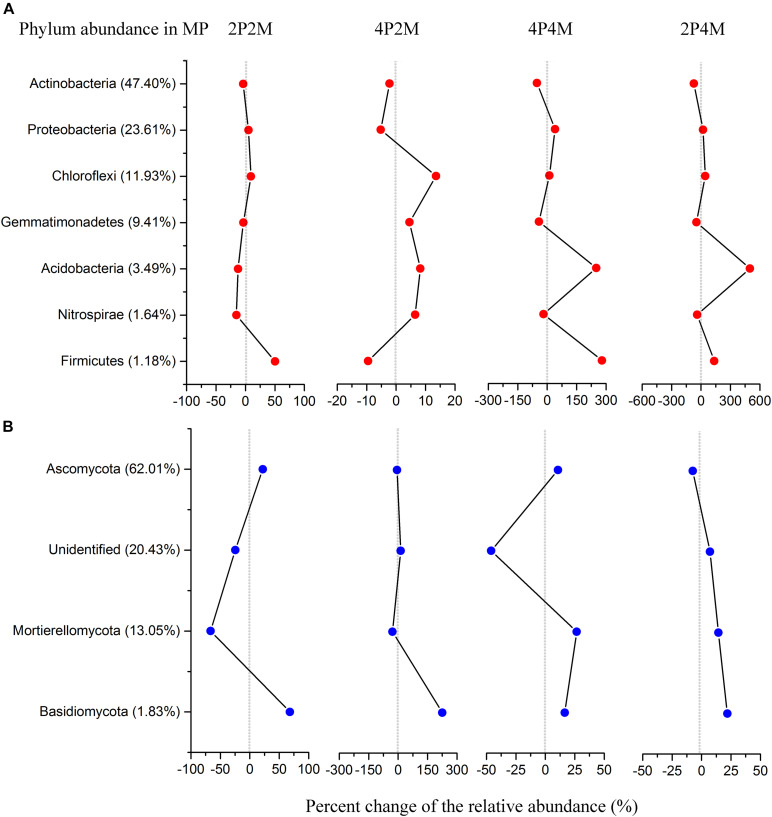
Distribution of bacterial **(A)** and fungal **(B)** phylum communities under the intercropping patterns. MP, 2P2M, 4P2M, 4P4M, and 2P4M represent the monoculture proso millet, 2 rows of proso millet intercropped with 2 rows of mung bean (2P2M), 4 rows of proso millet intercropped with 2 rows of mung bean (4P2M), 4 rows of proso millet intercropped with 4 rows of mung bean (4P4M), and 2 rows of proso millet intercropped with 4 rows of mung bean (2P4M), respectively.

The relative abundance of the soil bacterial communities showed seven predominant phyla (> 1%), namely, Actinobacteria (35.1%), Proteobacteria (26.5%), Chloroflexi (13.7%), Gemmatimonadetes (7.8%), Acidobacteria (8.5%), Firmicutes (2.2%) and Nitrospirae (1.4%) (Figure 5A and Supplementary Table 1). Among the microorganisms, the abundance of Proteobacteria was significantly increased under the 4P4M and 2P4M treatments. However, the average abundance of Actinobacteria was lower under the 4P4M and 2P4M treatments than the MP. Other phyla, such as Acidobacteria, Gemmatimonadetes, Chloroflexi, Nitrospirae and Firmicutes, were also altered by the cropping systems. Moreover, taxonomic classification showed that Actinobacteria was the most critical class and exhibited significant reductions under the 2P4M intercropping treatment (*P* < 0.05) (Supplementary Figure 3a and Supplementary Table 1). However, in Subgroup_6, the abundance of Acidobacteria was greater under the different intercropping patterns than the MP treatment. Further taxonomic classification showed that all principal bacterial groups at the order level (Supplementary Figure 4a and Supplementary Table 1) as well as Sphingomonadales, Nitrosomonadales, Myxococcales and Xanthomonadales were significantly reduced under the different intercropping patterns (*P* < 0.05).

In terms of the fungal community composition in different intercropping systems across all samples (Figure 5B and Supplementary Table 2), the phyla Ascomycota, Mortierellomycota and Basidiomycota had average contributions of 64.5, 11.7, and 3.1%, respectively. In particular, the relative abundance of Basidiomycota was significantly higher in the intercropping patterns than the monoculture proso millet (*P* < 0.05) and could be ranked as 4P2M > 2P2M > 2P4M > 4P4M > MP. Conversely, Ascomycota and Mortierellomycota showed different change trends and were significantly affected by intercropping (*P* < 0.01). Within Ascomycota, the classes Sordariomycetes and Eurotiomycetes were the most abundant and had mean relative abundances of 32.06 and 15.63%, respectively (Supplementary Figure 3b and Supplementary Table 2). At the order level, the abundance of Microascales was significantly higher than that of Xylariales and Onygenales, and the abundance of Pezizales was significantly lower under intercropping than MP (*P* < 0.01) (Supplementary Figure 4b and Supplementary Table 2).

### Relationship Among Plant Properties, Soil N and Soil Microbial Structure

**FIGURE 6:**
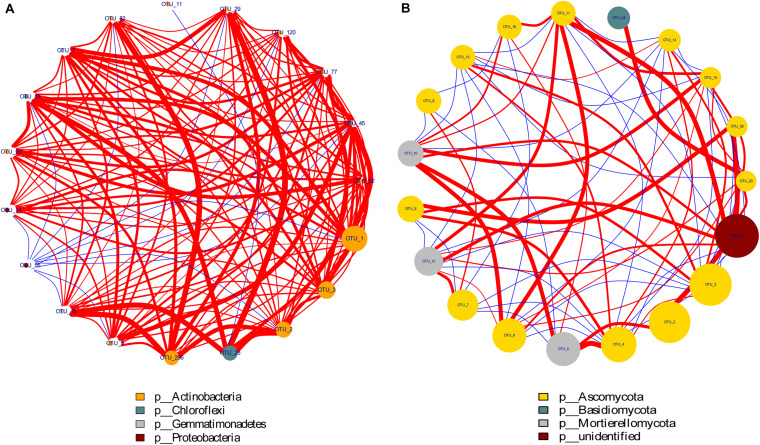
Network interaction diagram of dominant bacterial **(A)** and fungal **(B)** groups. Red lines indicate a positive correlation while the blue lines show a negative correlation. The size of the points represents the magnitude of phyla abundance while the thickness of the line represents the correlation size.

Through the Spearman test method, the top 20 most abundant OTUs of the bacterial and fungal phyla were selected for correlation analysis. As shown in Figure 6, the size of the points represents the magnitude of phyla abundance while the thickness of the line represents the correlation size. The red line indicated a positive correlation, and the blue line showed a negative correlation. The figure shows that there was a strong positive correlation among the top four dominant groups of bacteria, namely, included Proteobacteria, Gemmatimonadetes, Chloroflexi, and Actinobacteria. Moreover, the three dominant fungal communities Mortierellomycota, Basidiomycota, and Ascomycota also showed strong correlations. These organisms play a vital role in N cycling in soil-plant systems.

**FIGURE 7:**
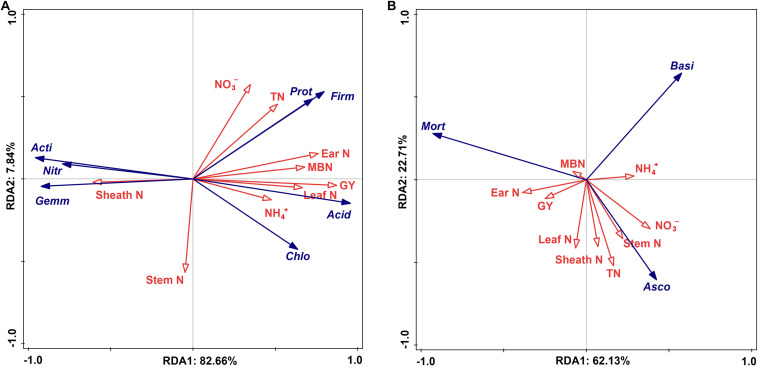
Ordination plots of the results from the redundancy analysis (RDA) to identify the relationships among the bacterial **(A)** and fungal **(B)** taxa (blue arrows) and the plant properties and soil N (red arrows) at the phylum level. Bacterial taxa: Actinobacteria (Acti), Proteobacteria (Prot), Chloroflexi (Chlo), Gemmatimonadetes (Gemm), Acidobacteria (Acid), Nitrospirae (Nitr), Firmicutes (Firm). Fungal taxa: Ascomycota (Asco), Mortierellomycota (Mort), Basidiomycota (Basi). Plant properties: Grain yield (GY), stem nitrogen (Stem N), leaf nitrogen (Leaf N), sheath nitrogen (Sheath N), ear nitrogen (Ear N). Soil N: Total nitrogen (TN), nitrate (NO_3_^–^-N), ammonium (NH_4_^+^–N), microbial biomass nitrogen (MBN).

The statistical analysis indicated that the bacterial alpha and beta diversities were markedly and positively related to the soil NO_3_^–^–N, grain weight per plant, and grain yield, whereas only the fungal alpha diversity showed similar positive correlations (Table 4). Moreover, a RDA was conducted to quantify the relative influence of environmental parameters (four soil N properties, namely, TN, NO_3_^–^–N, NH_4_^+^–N and MBN; and five plant properties, namely, stem N, leaf N, sheath N, ear N, and GY) on the microbial community composition at distinct taxonomic levels (phylum, class and order) (Figure 6 and Supplementary Figures 5, 6). The results showed that the environmental variables significantly affected the microbial community. In particular, TN and NO_3_^–^–N were significantly associated with the changes in Proteobacteria and Firmicutes while NH_4_^+^–N and MBN were significantly associated with the changes in Chloroflexi and Acidobacteria, respectively. Except for the stem N and sheath N, other plant properties were significantly and negatively associated with the abundance of Actinobacteria, Gemmatimonadetes and Nitrospirae at the bacterial phylum level (Figure 7A). For Proteobacteria, the soil properties and plant properties were correlated with the changes in the abundance of Sphingomonadales, Nitrosomonadales, and Xanthomonadales (Supplementary Figure 6a), which belong to Alphaproteobacteria, Betaproteobacteria, and Gammaproteobacteria, respectively (Supplementary Table 1). Moreover, Figure 7B shows that TN, NO_3_^–^–N and MBN among the soil properties and stem N, and GY among the plant properties influenced the abundance of Ascomycota and Mortierellomycota. For Ascomycota, the abundance of the class Pezizomycetes and the orders Mortierellales, Glomerellales and Sordariales orders were sensitive to changes in soil and plant properties (Supplementary Figures 5b, 6b).

**TABLE 4:** Spearman’s rank correlation coefficients (*R*) between microbial diversity (i.e., alpha diversity: Shannon index; beta diversity: PCoA) and plant properties and soil N.

Item	Bacteria		Fungi	
	alpha diversity (Shannon)	beta diversity (PCoA)	alpha diversity (Shannon)	beta diversity (PCoA)
Total nitrogen	0.414	0.518*	−0.836**	−0.612**
Nitrate	0.456*	0.554*	−0.847**	−0.616**
Ammonium	–0.137	–0.045	–0.126	–0.202
Microbial biomass nitrogen	0.160	−0.485*	0.178	−0.459*
Stem nitrogen	0.334	–0.239	0.268	–0.219
Leaf nitrogen	0.595**	–0.363	0.527*	–0.220
Sheath nitrogen	−0.452*	–0.011	−0.522*	–0.190
Ear nitrogen	0.634**	–0.344	0.607**	0.010
Ear number per plant	0.585**	–0.374	0.578**	–0.142
Ear length	0.129	–0.066	0.067	0.063
Grain weight per plant	0.778**	−0.576**	0.737**	–0.299
1000–grain weight	0.617**	–0.359	0.580**	–0.181
Grain yield	0.767**	−0.514*	0.721**	–0.249

*Correlation is significant at the 0.05 level; **Correlation is significant at the 0.01 level.

## Discussion

### Effect of Intercropping on Crop Grain Yield

Positive interactions occur in complex symbiotic systems that enhance the growth of crops, especially in cereal–legume intercropping systems, and these interactions are beneficial for improving soil fertility and yield (Hauggaard-Nielsen et al., 2001; Qian et al., 2018). In this study, intercropping led to a significant improvement in proso millet grain yield and variations in the ear number per plant, ear length, grain weight per plant and 1000-grain weight, and the grain yield increasing by 5.6–20.7% in 2017, 7.9–53.9% in 2018, and 28.3–75.4% in 2019 (Table 1). In contrast, the grain yield of intercropped mung bean were lower than those under the MM treatment at 34.8–55.8% in 2017, 34.4–38.8% in 2018, and 26.5–46.5% in 2019 (Table 2). These results could be explained by the spatial canopy structure, which resulted in greater light capture for the taller proso millet and less incident radiation for the shorter mung bean (Gong et al., 2020b). In intercropping systems, two species compete for light and their competitive abilities shift with their niches. For the production performance of intercropped proso millet, the greater light area and intensity from the margin effects were directly increased under intercropping by the combination of tall and short species, resulting in an advantage of light energy utilization (Wang et al., 2015); moreover, these helpful effects might facilitate interactions among the soil environment (such as the nutrient content), microbes and plants (Zhao et al., 2017). This theory is supported by the close relationship among the soil microbial composition, soil biochemical parameters, plant N and grain yield. Such improvements are caused by the N_2_ fixation of legumes, which maximizes and sustains plant growth for the intercropped proso millet.

### Effect of Intercropping on N Assimilation in the Soil–Plant System

Agricultural practices (i.e., tillage, cultivation, and fertilization) modify soil physicochemical properties and consequently alter soil nutrient conditions (Zhou et al., 2011; Jia et al., 2020). The directions and magnitudes of such differential responses are related to soil N because N is essential for plant growth and greatly affected by land use changes (Yang et al., 2017). The present results found that the TN, NO_3_^–^–N, NH_4_^+^–N and MBN contents were significantly increased under the intercropping systems compared with the monoculture systems, including those of proso millet (Figure 1) and mung bean (Figure 2). This finding may be attributed to the complex biological diversity under intercropping systems that results in the transfer of N to soil via ions and root exudates and further facilitates the accumulation and decomposition of soil N fractions. In legume–mixed intercropping, legumes increase N_2_ fixation to provide higher N levels for the N utilization of the adjacent crop, thus yielding a growth advantage for the intercropped plant (Subedi and Ma, 2005; Zhao et al., 2017). Interestingly, N is an important resource for cereals and the N fixed by legumes can be used by intercropped cereals during their growing period (Shen and Chu, 2004). Soil N fractions are presented as N-containing compounds, such as nucleic acids, amino sugars, and amino acids, which vary with the root structure, nutritional quality, and litter deposition. Therefore, based on the significant effect of interspecific interactions on N uptake under intercropping, proso millet–mung bean intercrops increase the soil N supply following mung bean N_2_ fixation in the rhizosphere. Moreover, soil microbes may change N recycling and utilization processes and could transform N to many different N forms, including mobile forms, to promote plant N assimilation (Vidal et al., 2019). Duchene et al. (2017) observed that rhizosphere N availability was influenced by intricate microbial activities under intercropping treatments. Increases in soil N forms, such as TN, NO_3_^–^–N, NH_4_^+^–N and MBN, could be produced via the high activity of N–fixing bacteria and ammonia–oxidizing bacteria under intercropping systems (Ramirez et al., 2012). Furthermore, belowground interspecific interactions may influence aboveground nutrient accumulation in cereal–legume intercropping systems. Compared with the N contents of monoculture crops, under the intercropping patterns, the average N contents in the stem, leaf, sheath, and ear tissues of proso millet increased by 28.6, 15.2, 16.7, and 12.0%, respectively, while the average N contents in the stem, leaf, petiole, and pod tissues of mung bean increased by 19.3, 13.0, 18.2, and 15.4%, respectively. This phenomenon indicated that higher soil nutrients may be expected to result in higher plant nutrients, which in turn results in the exudation of a more diverse range of organic compounds into the soil, thereby facilitating N assimilation. Additionally, it is generally accepted that belowground microbial communities can affect aboveground plant nutrient conditions by carrying out a wide spectrum of decomposition and metabolic processes. Thus, these findings indicated that intercropping could produce a large amount of N in rhizosphere soil and increase nutrient accumulation. Moreover, the habitats are fragile and the soil is barren and N limited on the Loess Plateau. Thus, intercropping systems alleviated N limitation and improved soil fertility in the present study.

### Intercropping Altered the Soil Microbial Diversity and Community to Facilitate N Assimilation

The valuable influences of intercropping in ameliorating biodiversity, heightening farmland ecosystem balance, and diminishing the occurrence of harmful organisms have been well documented (LaMondia et al., 2002; Ning et al., 2017). The soil microbial community is a fundamental component of soil quality and is imperative for many ecological processes, such as energy flow, nutrient cycling, and organic matter turnover (Acosta-Martínez et al., 2008). Our sequencing results showed that intercropping increased the soil bacterial alpha diversity (Shannon index), especially under the 4P4M and 2P4M treatments (Figure 4A), which pointed to the invulnerable soil microbes in this agricultural ecosystem. The Shannon index accounts for species richness abundance and evenness of the species present in the sample (Shannon, 1948). Thus, the greater increase in alpha diversity under intercropping suggested that rare species might have been more abundant. The changes in the bacterial community could affect soil N availability, and this conclusion was verified by the significant positive effect of NO_3_^–^–N on bacterial alpha diversity (Table 4). Soil microorganisms are pivotal players in driving biogeochemical cycles (Palomo et al., 2016); thus, the increased microbial diversity and N under intercropping would influence ecosystem processes and functions. Microbial biomass residues have been identified as significant sources of soil organic matter (Simpson et al., 2007; Miltner et al., 2012). The increase in soil microbial diversity and N may influence both the pool size and the chemical composition of soil carbon (Liang et al., 2011). In addition, links between microbial diversity and plant productivity, plant diversity, or nutrient acquisition have also been observed (Zak et al., 2003). Soil fungal alpha diversity was less affected by intercropping patterns compared with the bacteria, which may have been caused by the number of copiotrophic characteristics of bacteria (Ramirez et al., 2012). The nutrient supply under intercropping systems was more abundant than that under traditional monocultures and could provide more sufficient nutrients for bacterial growth. Although Ren et al. (2017b) found that the soil bacterial and fungal community characteristics were affected by altitude, they showed that soil bacterial alpha diversity fluctuated more along altitudinal gradients than fungal alpha diversity, which was likely because of soil N. N metabolism in soil is overwhelmingly associated with bacterial activity. Thus, a greater abundance of bacteria in the intercropping system illustrates that bacteria may play a crucial role in facilitating N metabolism in the rhizosphere. Compared with monocots, dicots, especially legumes, generate and secrete more organic compounds into the crop rhizosphere (Raghothama, 1999). Importantly, increases in the organic compound content in root exudates can provide more carbon sources for rhizosphere microorganism growth, which may be another reason for the change in the bacterial community in the intercropping treatments. These results illustrated that the intercropping treatment had some valuable effects on soil microbial activity, which in turn could facilitate the nutrient supply for plants as previously reported (Hao et al., 2003).

Microbial communities play an active role in the maintenance of soil functions in ecosystem processes (Van Der Heijden et al., 2008), such as decomposing organic matter and transforming inorganic N to organic N (Leiros et al., 2000; Prosser and Nicol, 2008). The soil bacterial community compositions revealed that the 4P4M and 2P4M treatments significantly increased the abundance of the phylum Proteobacteria. However, although Actinobacteria was the most abundant bacterial phylum, the average abundance of Actinobacteria was lower with intercropping under the same conditions compared with that under MP (Figure 5A). The change in the abundance of bacteria may be in connection with the habitat for low-nutrient consumption groups that are well adapted to unstable N levels in the soil (Stach and Bull, 2005). Chen et al. (2003) found that N fixation by Betaproteobacteria in the legume symbiotic system is widespread in nature. Our study revealed that TN and NO_3_^–^–N were significantly positively related to Proteobacteria (Figure 7A). Consequently, N assimilation was enhanced by Proteobacteria. Furthermore, Sphingomonadales and Xanthomonadales are members of the Alphaproteobacteria and Gammaproteobacteria classes (Supplementary Table 1) and heterotrophic and N–fixing organisms. Sphingomonadales are also considered rhizosphere plant–promoting bacteria and can fix atmospheric N_2_ in symbiosis with plants (Regupathy et al., 2012). Hence, the increase in the abundance of Proteobacteria was beneficial for N accumulation and cycling. Moreover, other bacterial taxa, such as Gemmatimonadetes and Nitrospirae, exhibited strong responses to changes in plant N and soil N, and the changes in these two phyla were likely due to their own ecological characteristics (Ivanova et al., 2016). Zhang et al. (2015) observed that the abundance of ammonia oxidizers decreases in the crop rhizospheres under maize–faba bean intercropping, and this finding indirectly supports our explanation. The positive correlations among the dominant bacterial communities are shown in Figure 6A and indicate that under suitable conditions, these groups grow together to ensure their individual and collective survival and dominance over most other organisms in the soil. Altogether, proso millet intercropped with mung bean greatly affected the dominant bacterial communities, and the functions of these communities can provide feedback to facilitate N assimilation.

For the soil fungal community composition, the abundances of both Ascomycota and Mortierellomycota were 75% (Figure 5B), which is consistent with reports of these taxa on a global scale for arid farmlands (Li and Wu, 2018). However, these two dominant phyla were not responsive to environmentally induced changes in intercropping conditions and only Ascomycota was affected by TN and NO_3_^–^–N (Figure 7B). This phenomenon could be explained by the functions of Ascomycota. The abundance of Ascomycota was increased by residue degradation, and these fungi can rapidly metabolize organic substrates of rhizodeposition in rhizosphere soil (Bastida et al., 2013). Hence, proso millet–mung bean intercropping may create suitable circumstances for Ascomycota and allow them to better exploit the easily degradable fraction of plant residues and facilitate N accumulation. Moreover, the growth rates of Ascomycota were increased by N availability, thereby expediting the decomposition of plant residues (Fontaine et al., 2011). In addition, according to the growth rate of the microorganisms (GRH; Elser et al., 1996; Ren et al., 2017a), the growth of most species is associated with a greater demand for N for the synthesis of ribosomal DNA and protein. Therefore, the relative abundance of this dominant microbial phylum was very sensitive to changes in soil N dynamics and availability. However, at the order level, Hypocreales (a member of Sordariomycetes in Ascomycota), which is related to the soil N fractions and plant N accumulation (Supplementary Figure 6), showed significant decreasing trends under the intercropping pattern (Supplementary Table 2) because Hypocreales is a rapidly growing plant tissue decomposer (Hannula et al., 2012) and the lower amount of litter at the proso millet flowering stage under intercropping led to a decrease in the abundance of this community. Moreover, studies have also shown that many species belonging to the order Hypocreales are insect pathogens (Castrillo et al., 2008; Riosvelasco et al., 2014) and increasing their abundance would contribute to the control of pests and diseases under field conditions. Furthermore, combined with the composition of the bacterial community, these discoveries illustrated that the microbial diversity and community composition (bacterial and fungal) in rhizosphere soil were altered under proso millet/mung bean intercropping, which promoted plant N assimilation and increased grain yield.

## Conclusion

Our data clearly indicated that proso millet/mung bean intercropping altered the abundance of soil bacteria and fungi. The soil bacterial alpha diversity was higher under intercropping patterns and varied more than the fungal alpha diversity. With regard to the bacterial composition, the phylum Proteobacteria, which is positively related to soil N accumulation, significantly increased. The changes in the composition of Proteobacteria, such as in the orders Sphingomonadales and Xanthomonadales, influenced N assimilation. For the fungal community composition, Ascomycota was the principal phylum and was affected by TN and NO_3_^–^–N. Microbial changes enhanced N uptake in the soil and promoted N accumulation in different plant organs, resulting in an improvement in grain yield. The average grain yield under the 2P4M intercropping treatment increased by 50.1% compared with that of the monoculture proso millet, showing the highest productivity. Our study shows that the intercropping of proso millet with mung bean influenced the microbial (bacterial and fungal) communities and provides insight into the roles of microbial biodiversity and ecological performance in improving crop production.

## Data Availability Statement

Sequence data associated with this project have been deposited into the National Center for Biotechnology Information (NCBI) (accession numbers of PRJNA669229 for bacteria and PRJNA670554 for fungi).

## Author Contributions

KD and XG performed the experiments and drafted the manuscript. AI and BF designed the project and guided the experiments. GZ and HW investigated the material characteristics. KD and XG analyzed the data and planted the material. BF organized and coordinated the whole project. All authors contributed to the article and approved the submitted version.

## Conflict of Interest

The authors declare that the research was conducted in the absence of any commercial or financial relationships that could be construed as a potential conflict of interest.
