# Supplementary material for: Intercropping Alters the Soil Microbial Diversity and Community to Facilitate Nitrogen Assimilation: A Potential Mechanism for Increasing Proso Millet Grain Yield
Source: Front Microbiol. 2020 Nov 24;11:601054. doi: 10.3389/fmicb.2020.601054 (PMC7721675; doi:10.3389/fmicb.2020.601054)
Supplement: Supplementary file 1 [file Data_Sheet_1.docx]

**TABLE S1**

Relative abundances (average values and standard error) of bacterial compositions across taxonomical classification (Phyla, Class, and Order) under intercropping patterns. Different letters indicate significant differences (*P* < 0.05) among different intercropping patterns.

| ***Phyla*** | ***Class*** | ***Order*** | **MP** | **2P2M** | **4P2M** | **4P4M** | **2P4M** | **F** | ***P*** |
| --- | --- | --- | --- | --- | --- | --- | --- | --- | --- |
| ***Acti*** |  |  | 47.40±0.72a | 45.59±3.54a | 46.35±1.80a | 22.87±5.72b | 13.41±1.32c | 98.324 | <0.001 |
|  | *Actin* |  | 34.65±0.96a | 33.26±3.46a | 33.30±2.13a | 14.49±2.68b | 9.57±1.00c | 112.990 | <0.001 |
|  |  | *Microc* | 15.78±0.73a | 14.59±3.45a | 14.47±2.14a | 7.12±1.12b | 5.25±0.61b | 25.495 | <0.001 |
|  |  | *Franki* | 6.17±0.28a | 5.74±0.39a | 6.05±0.24a | 1.85±0.87b | 0.67±0.14c | 128.221 | <0.001 |
|  |  | *Propio* | 4.35±0.52a | 3.91±0.33a | 4.05±0.21a | 1.77±0.28b | 1.42±0.27b | 65.78 | <0.001 |
|  |  | *Strept* | 2.46±0.24b | 2.89±0.19a | 2.96±0.22a | 1.03±0.35c | 0.75±0.17c | 76.794 | <0.001 |
|  |  | *Microm* | 2.02±0.15a | 2.01±0.20a | 2.17±0.33a | 0.58±0.13b | 0.45±0.23b | 61.145 | <0.001 |
|  |  | *Pseudo* | 1.51±0.19a | 1.35±0.11a | 1.55±0.13a | 0.65±0.16b | 0.31±0.07c | 65.574 | <0.001 |
|  |  | *Coryne* | 1.08±0.09b | 1.39±0.11a | 1.04±0.12b | 0.72±0.02c | 0.29±0.10d | 79.175 | <0.001 |
|  | *Acidi* |  | 7.26±0.26a | 7.36±0.44a | 7.30±0.26a | 4.37±1.78b | 1.83±0.07c | 35.091 | <0.001 |
|  |  | *Acidim* | 7.26±0.26a | 7.36±0.44a | 7.30±0.26a | 4.37±1.78b | 1.83±0.07c | 35.091 | <0.001 |
|  | *Thermol* |  | 4.29±0.39a | 3.76±0.14ab | 4.50±0.42a | 3.32±1.13b | 1.28±0.30c | 19.179 | <0.001 |
|  |  | *Soliru* | 2.60±0.38a | 2.41±0.18a | 2.46±0.28a | 1.98±0.73a | 0.66±0.22b | 15.314 | <0.001 |
|  |  | *Gaiell* | 1.68±0.12b | 1.35±0.17b | 2.04±0.18a | 1.35±0.41b | 0.62±0.16c | 20.401 | <0.001 |
|  | *MB–A2–108* |  | 0.96±0.12a | 0.96±0.24a | 1.02±0.07a | 0.54±0.15b | 0.60±0.34b | 4.754 | 0.011 |
| ***Prot*** |  |  | 23.61±0.72c | 24.81±1.55c | 22.38±0.75c | 33.06±1.67a | 28.60±3.61b | 20.237 | <0.001 |
|  | *Alpha* |  | 15.28±0.68a | 16.39±1.03a | 13.92±0.59b | 16.26±0.57a | 13.97±1.10b | 8.380 | <0.001 |
|  |  | *Rhizob* | 8.89±0.75ab | 9.99±0.70a | 8.39±0.65b | 9.42±0.64ab | 7.27±0.87c | 8.173 | <0.001 |
|  |  | *Rhodos* | 4.06±0.41a | 3.86±0.44a | 3.51±0.31ab | 3.06±0.38b | 2.39±0.44c | 11.414 | <0.001 |
|  |  | *Sphing* | 1.60±0.11c | 1.55±0.11c | 1.46±0.12c | 2.06±0.27b | 2.96±0.31a | 37.460 | <0.001 |
|  | *Betap* |  | 4.56±0.30ab | 1.29±0.30b | 4.84±0.38ab | 4.99±0.44ab | 5.26±0.88a | 2.189 | 0.120 |
|  |  | *Nitroso* | 2.91±0.39a | 2.76±0.21a | 3.07±0.43a | 2.60±0.37a | 2.52±0.56a | 1.225 | 0.342 |
|  | *Delta* |  | 2.55±0.30c | 2.97±0.34c | 2.51±0.21c | 6.14±1.00a | 4.39±1.04b | 20.772 | <0.001 |
|  |  | *Myxoco* | 1.88±0.26c | 2.32±0.29bc | 1.79±0.16c | 4.84±0.92a | 3.11±0.91b | 17.188 | <0.001 |
|  | *Gamma* |  | 1.21±0.20b | 1.14±0.07b | 1.10±0.14b | 5.63±0.20a | 4.95±1.07a | 83.775 | <0.001 |
|  |  | *Xantho* | 1.04±0.14b | 0.98±0.09b | 1.00±0.14b | 4.51±0.20a | 4.00±0.98a | 61.105 | <0.001 |
| ***Chlo*** |  |  | 11.93±1.03b | 12.93±1.18b | 13.50±1.27b | 13.28±2.06b | 16.93±2.09a | 5.283 | <0.001 |
|  | *Thermom* |  | 5.29±0.94ab | 6.03±1.17a | 6.26±1.02a | 2.82±0.88c | 3.96±0.60bc | 9.616 | <0.001 |
|  |  | *JG30–KF–CM45* | 4.62±0.85a | 5.14±1.07a | 5.44±0.89a | 2.27±0.67b | 3.14±0.45b | 11.181 | <0.001 |
|  | *KD4–96* |  | 1.70±0.25ab | 1.71±0.48ab | 2.10±0.28a | 1.46±0.22b | 1.87±0.39ab | 1.937 | 0.157 |
|  | *Chlor* |  | 1.37±0.05a | 1.25±0.26a | 1.40±0.11a | 1.49±0.46a | 1.39±0.41a | 0.336 | 0.850 |
|  | *TK10* |  | 1.18±0.13bc | 1.05±0.12c | 1.13±0.10c | 1.43±0.08a | 1.35±0.21ab | 5.460 | <0.001 |
| ***Gemm*** |  |  | 9.41±0.67a | 9.05±0.41a | 9.83±0.70a | 5.40±1.16b | 5.07±0.75b | 35.392 | <0.001 |
|  | *Gemma* |  | 8.48±0.64a | 7.92±0.30a | 8.86±0.73a | 4.19±0.80b | 3.81±0.57b | 60.209 | <0.001 |
|  |  | *Gemmat* | 8.48±0.64a | 7.92±0.30a | 8.86±0.73a | 4.19±0.80b | 3.81±0.57b | 60.209 | <0.001 |
| ***Acid*** |  |  | 3.49±0.39c | 3.00±0.37c | 3.72±0.29c | 11.72±4.82b | 20.57±2.97a | 35.466 | <0.001 |
|  | *Subgroup_6* |  | 1.36±0.35c | 1.28±0.18c | 1.64±0.26c | 6.99±3.82b | 13.88±2.04a | 31.949 | <0.001 |
| ***Nitr*** |  |  | 1.64±0.18a | 1.38±0.07b | 1.74±0.15a | 1.33±0.22b | 1.00±0.20c | 11.499 | <0.001 |
|  | *Nitro* |  | 1.64±0.18a | 1.38±0.07b | 1.74±0.15a | 1.33±0.22b | 1.00±0.20c | 11.499 | <0.001 |
|  |  | *Nitrosp* | 1.64±0.18a | 1.38±0.07b | 1.74±0.15a | 1.33±0.22b | 1.00±0.20c | 11.499 | <0.001 |
| ***Firm*** |  |  | 1.18±0.14d | 1.75±0.25c | 1.06±0.15d | 4.42±0.55a | 2.71±0.57b | 43.950 | <0.001 |

**Phyla level:** *Actinobacteria (Acti), Proteobacteria (Prot), Chloroflexi (Chlo), Gemmatimonadetes (Gemm), Acidobacteria (Acid), Nitrospirae (Nitr), Firmicutes (Firm).*

**Class level**: *Actinobacteria (Actin), Acidimicrobiia (Acidi), Thermoleophilia (Thermol),* *Alphaproteobacteria (Alpha), Betaproteobacteria (Betap), Deltaproteobacteria (Delta), Gammaproteobacteria (Gamma), Thermomicrobia (Thermom), Chloroflexia (Chlor), Gemmatimonadetes (Gemma), Nitrospira (Nitro).*

**Order Level**: *Micrococcales (Microc),* *Frankiales (Franki), Propionibacteriales (Propio), Streptomycetales (Strept), Micromonosporales (Microm), Pseudonocardiales (Pseudo), Corynebacteriales (Coryne), Acidimicrobiales (Acidim), Solirubrobacterales (Soliru), Gaiellales (Gaiell),* *Rhizobiales (Rhizob), Rhodospirillales (Rhodos),* *Sphingomonadales (Sphing),* *Nitrosomonadales (Nitroso),* *Myxococcales (Myxoco),* *Xanthomonadales (Xantho), Gemmatimonadales (Gemmat), Nitrospirales (Nitrosp).*

MP, 2P2M, 4P2M, 4P4M, and 2P4M represent the monoculture proso millet, 2 rows of proso millet alternated with 2 rows of mung bean, 4 rows of proso millet alternated with 2 rows of mung bean, 4 rows of proso millet alternated with 4 rows of mung bean, and 2 rows of proso millet alternated with 4 rows of mung bean, respectively.**TABLE S2**

Relative abundances (average values and standard error) of fungal compositions across taxonomical classification (Phyla, Class, and Order) under intercropping patterns. Different letters indicate significant differences (*P* < 0.05) among different intercropping patterns.

| ***Phyla*** | ***Class*** | ***Order*** | **MP** | **2P2M** | **4P2M** | **4P4M** | **2P4M** | **F** | ***P*** |
| --- | --- | --- | --- | --- | --- | --- | --- | --- | --- |
| ***Asco*** |  |  | 62.01±2.58c | 75.68±1.55a | 58.62±1.86d | 68.70±1.32b | 57.39±2.86d | 52.092 | <0.001 |
|  | *Sorda* |  | 30.23±0.65b | 38.75±0.85a | 38.29±1.76a | 29.61±1.89b | 23.42±1.87c | 74.371 | <0.001 |
|  |  | *Hypocr* | 11.44±1.40a | 4.06±0.31c | 11.68±0.68a | 6.91±0.64b | 10.24±2.36a | 25.533 | <0.001 |
|  |  | *Sordar* | 5.68±1.10a | 2.32±0.38c | 6.40±0.24a | 3.37±0.21b | 3.51±0.46b | 94.951 | <0.001 |
|  |  | *Microa* | 3.40±0.61d | 25.83±1.50a | 5.40±0.07c | 15.64±1.79b | 4.87±0.38cd | 24.093 | <0.001 |
|  |  | *Xylari* | 1.60±0.62a | 0.04±0.01b | 0.17±0.05b | 0.06±0.02b | 0.02±0.00b | 17.452 | <0.001 |
|  |  | *Glomer* | 1.21±0.09b | 1.12±0.22b | 2.66±0.34a | 0.52±0.06d | 0.82±0.05c | 75.837 | <0.001 |
|  | *Eurot* |  | 18.46±2.85a | 15.45±0.97a | 9.47±0.45b | 18.14±1.87a | 16.64±2.45a | 14.207 | <0.001 |
|  |  | *Onygen* | 18.28±2.85a | 15.33±0.99a | 9.11±0.40b | 17.97±1.86a | 16.45±2.43a | 14.916 | <0.001 |
|  | *Leoti* |  | 3.31±0.81a | 0.59±0.16c | 1.75±0.49b | 2.65±0.33a | 2.82±0.47a | 18.470 | <0.001 |
|  |  | *Leotio* | 3.12±0.78a | 0.55±0.15d | 1.60±0.49c | 2.30±0.23bc | 2.56±0.44ab | 17.452 | <0.001 |
|  | *Peziz* |  | 2.89±0.47a | 1.41±0.94b | 1.66±0.59b | 1.19±0.53b | 1.33±0.44b | 4.896 | <0.001 |
|  |  | *Peziza* | 2.66±0.54a | 1.37±0.95b | 1.60±0.58b | 0.83±0.12b | 1.19±0.42b | 5.540 | <0.001 |
| ***Mort*** |  |  | 13.05±0.77c | 4.37±0.48e | 9.36±1.06d | 16.53±0.40a | 14.93±1.64b | 98.438 | <0.001 |
|  | *Morti* |  | 12.49±0.57c | 4.33±0.48e | 9.31±1.07d | 16.43±0.39a | 14.90±1.64b | 102.692 | <0.001 |
|  |  | *Mortie* | 12.49±0.57c | 4.33±0.48e | 9.31±1.07d | 16.43±0.39a | 14.90±1.64b | 102.692 | <0.001 |
| ***Basi*** |  |  | 1.83±0.45b | 3.07±1.51b | 5.94±0.24a | 2.13±0.31b | 2.23±0.84b | 17.134 | <0.001 |
|  | *Agari* |  | 1.32±0.38c | 2.90±0.73b | 5.66±0.17a | 1.92±0.33c | 2.05±0.72c | 43.879 | <0.001 |

**Phyla level:** *Ascomycota (Asco),* *Mortierellomycota (Mort), Basidiomycota (Basi).*

**Class level**: *Sordariomycetes (Sorda),* *Eurotiomycetes (Eurot), Leotiomycetes (Leoti), Mortierellomycetes (Morti),* *Pezizomycetes (Peziz), Agaricomycetes (Agari).*

**Order Level**: *Onygenales (Onygen),* *Mortierellales (Mortie), Hypocreales (Hypocr),* *Sordariales (Sordar), Leotiomycetes (Leotio),* *Xylariales (Xylari), Microascales (Microa),* *Pezizales (Peziza),* *Glomerellales (Glomer).*

MP, 2P2M, 4P2M, 4P4M, and 2P4M represent the monoculture proso millet, 2 rows of proso millet alternated with 2 rows of mung bean, 4 rows of proso millet alternated with 2 rows of mung bean, 4 rows of proso millet alternated with 4 rows of mung bean, and 2 rows of proso millet alternated with 4 rows of mung bean, respectively.
