# Supplementary material for: Intercropping Alters the Soil Microbial Diversity and Community to Facilitate Nitrogen Assimilation: A Potential Mechanism for Increasing Proso Millet Grain Yield
Source: Front Microbiol. 2020 Nov 24;11:601054. doi: 10.3389/fmicb.2020.601054 (PMC7721675; doi:10.3389/fmicb.2020.601054)
Supplement: Supplementary file 2 [file Data_Sheet_2.docx]

**Supplementary information:**

**Intercropping Alters the Soil Microbial Diversity and Community to Facilitate Nitrogen Assimilation: A Potential Mechanism for Increasing Proso Millet Grain Yield**

***Ke Dang^1^****^†^****, Xi******angwei Gong^1^****^†^****, Guan Zhao^1^, Honglu Wang^1^, Aliaksandr Ivanistau^2^, Baili Feng******^1**^***

*^1^ College of Agronomy, State Key Laboratory of Crop Stress Biology in Arid Areas/**Northwest A & F University,* *Yangling, Shaanxi 712100, PR China*

*^2^ Belarusian State Agricultural Academy, 213407 Gorki, Mogilev Region, Belarus*

*^†^* **These authors contributed equally to this work.**

* Corresponding author: Dr. Baili Feng

E–mail: [fengbaili@nwsuaf.edu.cn](mailto:fengbaili@nwsuaf.edu.cn)

Phone: +86–29–87082889; Fax: +86–29–87082889


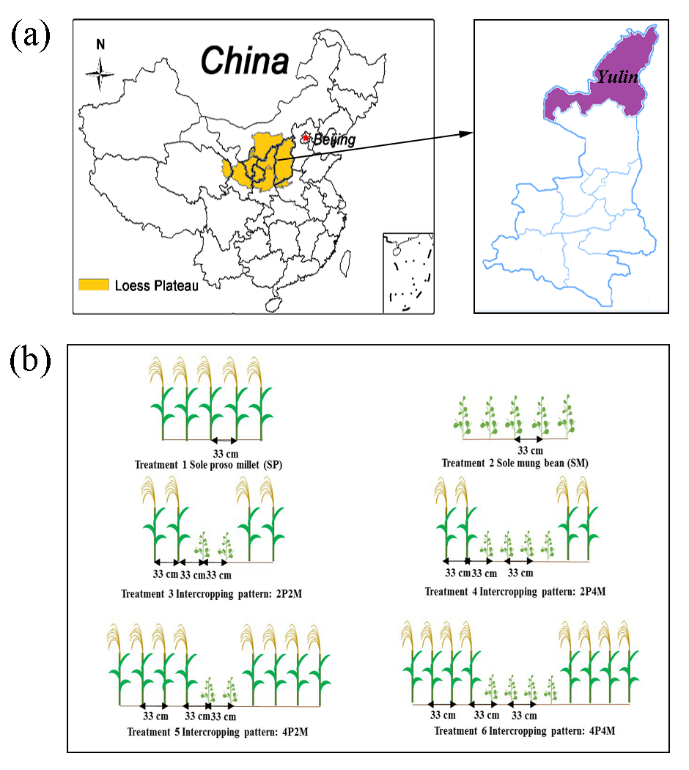


**FIGURE S1** Location of Yu’lin on the Loess Plateau of China (**a**) and schematic illustration of row placement in the different experimental planting patterns (**b**).


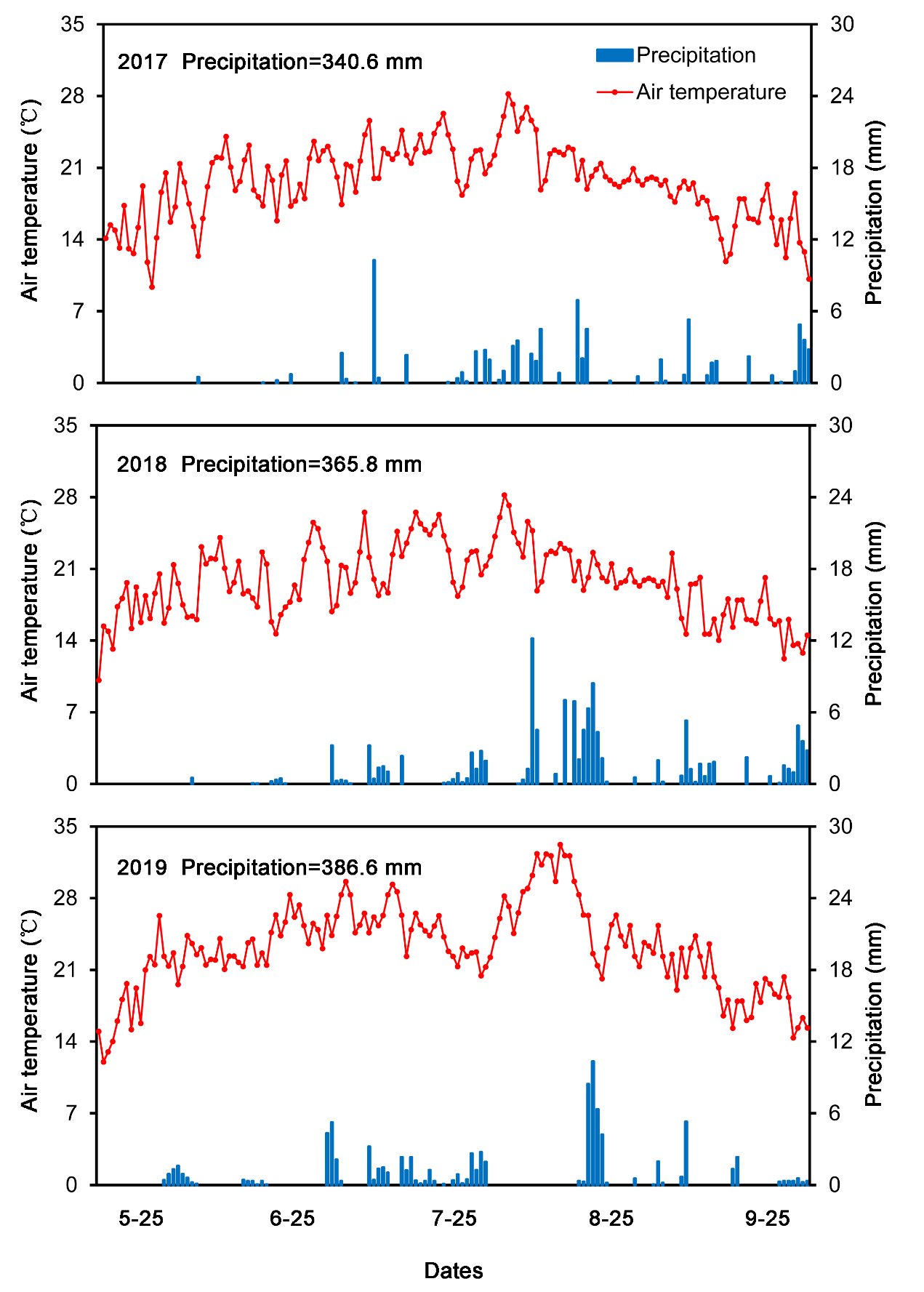


**FIGURE S2** Daily air temperature and precipitation during the crop growing season (May-September) in 2017, 2018 and 2019 in Yulin, Shaanxi, China


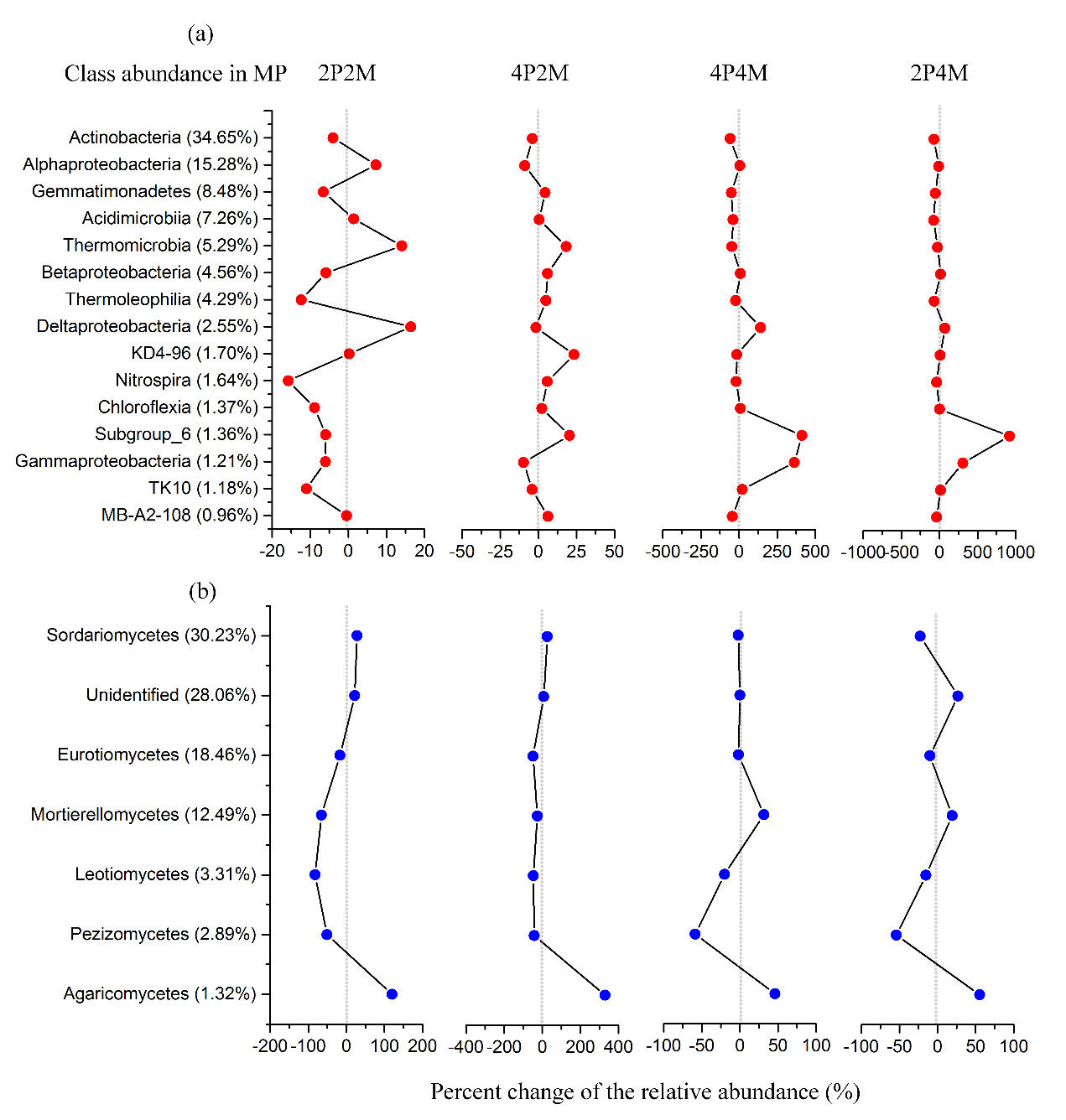


**FIGURE S3** Distribution of bacterial (**a**) and fungal (**b**) class communities under intercropping patterns. MP, 2P2M, 4P2M, 4P4M, and 2P4M represent the monoculture proso millet, 2 rows of proso millet intercropped with 2 rows of mung bean (2P2M), 4 rows of proso millet intercropped with 2 rows of mung bean (4P2M), 4 rows of proso millet intercropped with 4 rows of mung bean (4P4M), and 2 rows of proso millet intercropped with 4 rows of mung bean (2P4M), respectively.

**
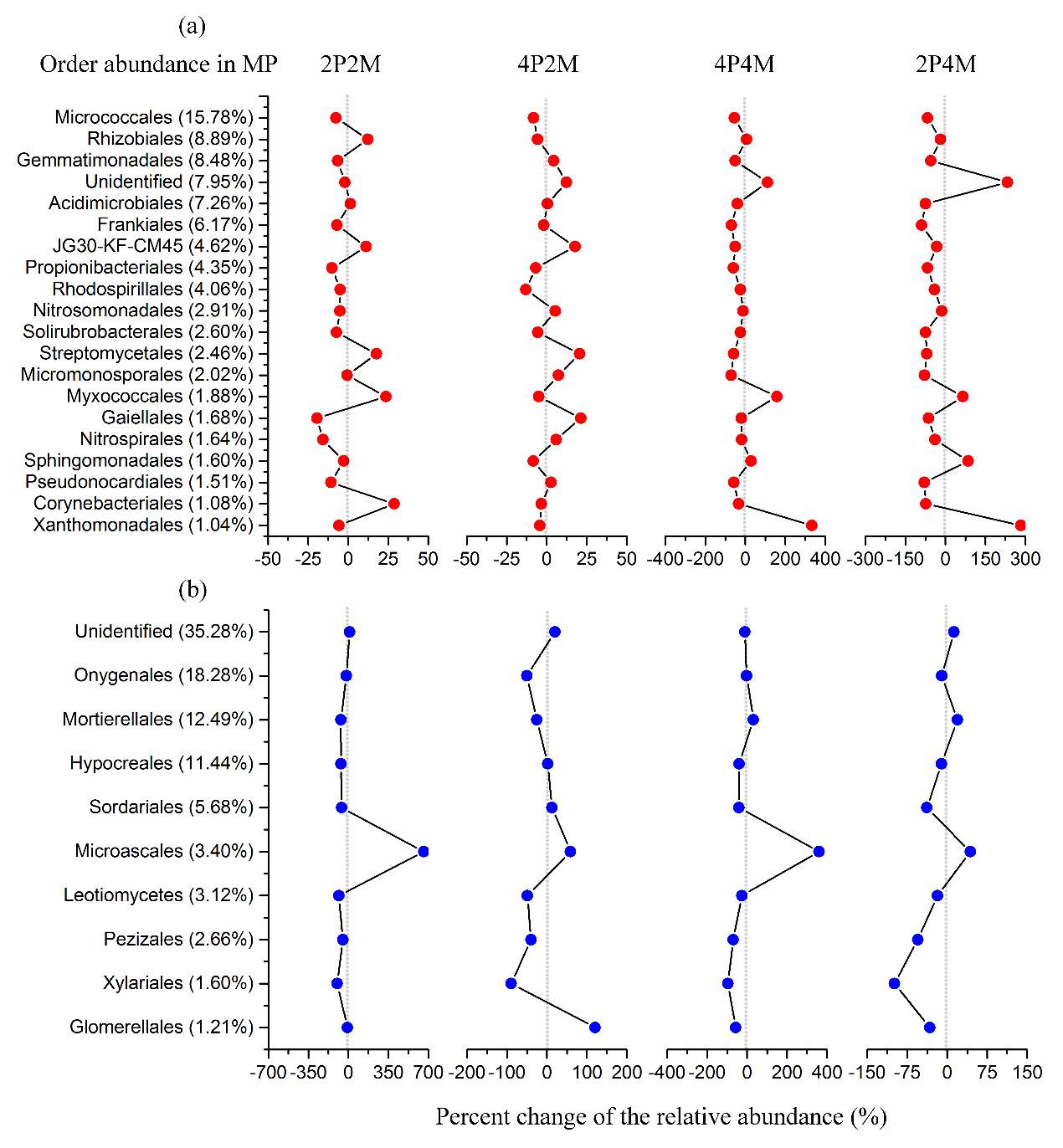
**

**FIGURE S4** Distribution of bacterial (**a**) and fungal (**b**) order communities under intercropping patterns. MP, 2P2M, 4P2M, 4P4M, and 2P4M represent the monoculture proso millet, 2 rows of proso millet intercropped with 2 rows of mung bean (2P2M), 4 rows of proso millet intercropped with 2 rows of mung bean (4P2M), 4 rows of proso millet intercropped with 4 rows of mung bean (4P4M), and 2 rows of proso millet intercropped with 4 rows of mung bean (2P4M), respectively.


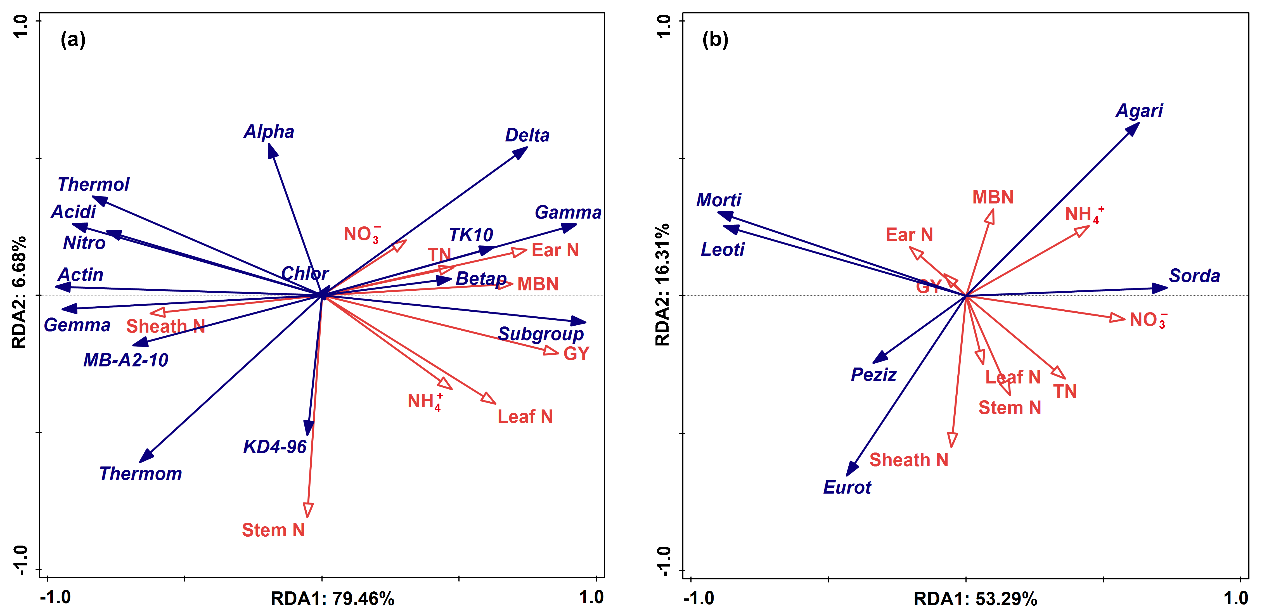


**FIGURE S5** Ordination plots of the results from the redundancy analysis (RDA) to identify the relationships among the bacterial (**a**) and fungal (**b**) taxa (blue arrows) and the plant properties and soil N (red arrows) at the Class level.

**Bacterial taxa**: *Actinobacteria (Actin),* *Acidimicrobiia (Acidi), Thermoleophilia (Thermol), Alphaproteobacteria (Alpha), Betaproteobacteria (Betap), Deltaproteobacteria (Delta), Gammaproteobacteria (Gamma), Thermomicrobia (Thermom), Chloroflexia (Chlor**), Gemmatimonadetes (Gemma),* *Nitrospira (Nitro).*

**Fungal taxa**: *Sordariomycetes (Sorda), Eurotiomycetes (Eurot), Leotiomycetes (Leoti), Mortierellomycetes (Morti), Pezizomycetes (Peziz), Agaricomycetes (Agari),* *Unidentified (Unide).*

**Plant properties:** Grain yield (GY), stem nitrogen (Stem N), leaf nitrogen (Leaf N), sheath nitrogen (Sheath N), ear nitrogen (Ear N).

**Soil N:** Total nitrogen (TN), nitrate (NO_3_^−^−N), ammonium (NH_4_^+^–N), microbial biomass nitrogen (MBN).

**
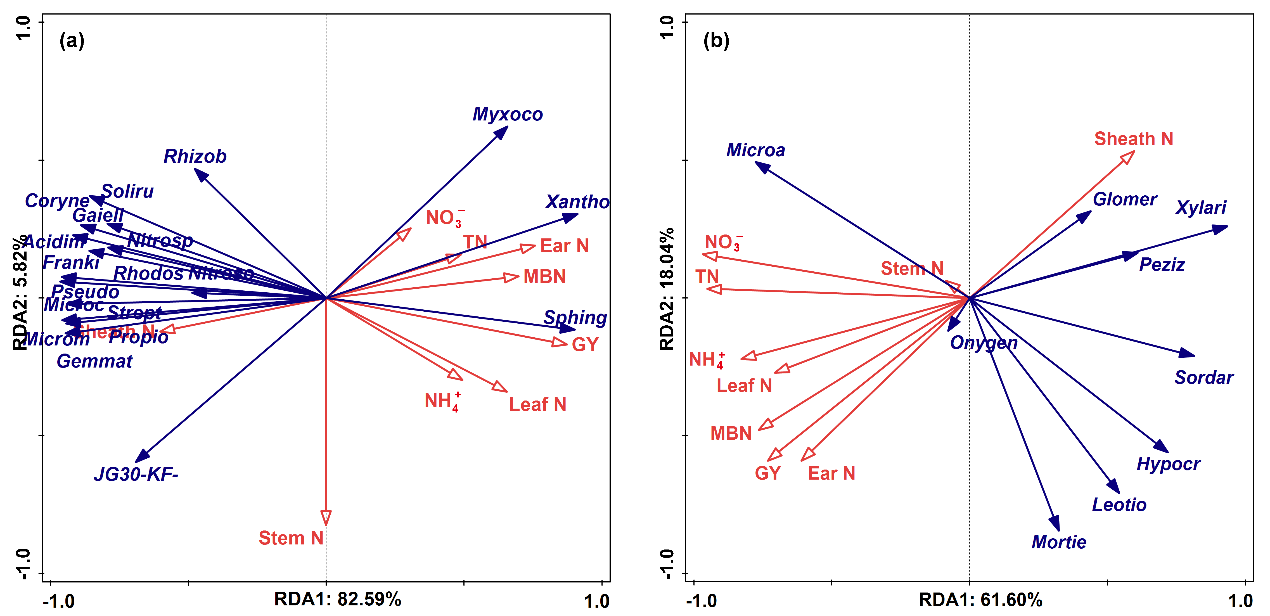
**

**FIGURE S6** Ordination plots of the results from the redundancy analysis (RDA) to identify the relationships among the bacterial (**a**) and fungal (**b**) taxa (blue arrows) and the plant nitrogen, soil nitrogen, plant biomass and grain yield (red arrows) at the Order level.

**Bacterial taxa**: *Micrococcales (Microc), Frankiales (Franki), Propionibacteriales (Propio), Streptomycetales (Strept), Micromonosporales (Microm), Pseudonocardiales (Pseudo), Corynebacteriales (Coryne), Acidimicrobiales (Acidim), Solirubrobacterales (Soliru), Gaiellales (Gaiell), Rhizobiales (Rhizob), Rhodospirillales (Rhodos), Sphingomonadales (Sphing), Nitrosomonadales (Nitroso), Myxococcales (Myxoco), Xanthomonadales (Xantho), Gemmatimonadales (Gemmat), Nitrospirales (Nitrosp),* *Unidentified (Uniden).*

**Fungal taxa**: *Onygenales (Onygen), Mortierellales (Mortie), Hypocreales (Hypocr), Sordariales (Sordar), Leotiomycetes (Leotio), Xylariales (Xylari), Microascales (Microa), Pezizales (Peziza), Glomerellales (Glomer), Unidentified (Uniden).*

**Plant properties:** Grain yield (GY), stem nitrogen (Stem N), leaf nitrogen (Leaf N), sheath nitrogen (Sheath N), ear nitrogen (Ear N).

**Soil N:** Total nitrogen (TN), nitrate (NO_3_^−^−N), ammonium (NH_4_^+^–N), microbial biomass nitrogen (MBN).
